# A Rare Presentation of Granulomatosis With Polyangiitis: A Case Report and Literature Review

**DOI:** 10.1002/ccr3.72941

**Published:** 2026-06-14

**Authors:** Anil Shahi, Ayush Neupane, Bishaka Adhikari

**Affiliations:** ^1^ Department of Internal Medicine Chitwan Medical College Bharatpur Nepal

**Keywords:** ANCA associated Vasculitis, diffuse alveolar hemorrhage, granulomatosis with polyangiitis, pancreatic mass

## Abstract

Granulomatosis with polyangiitis (GPA) is a rare ANCA‐associated necrotizing vasculitis that predominantly affects the respiratory tract and kidneys. Gastrointestinal involvement is uncommon, and pancreatic manifestations are exceedingly rare. Diffuse alveolar hemorrhage (DAH) represents a severe and potentially fatal complication. We report a 44‐year‐old male who was initially diagnosed with a pancreatic mass suspicious for malignancy and planned for pancreatectomy. During hospitalization, he developed acute respiratory distress with hemoptysis. Imaging revealed diffuse ground‐glass opacities consistent with DAH and a bulky pancreatic tail lesion. Laboratory investigations demonstrated severe anemia, urinary abnormalities suggestive of glomerulonephritis, and strongly positive c‐ANCA. A diagnosis of granulomatosis with polyangiitis was established based on clinical, radiological, and serological findings. The patient was treated with intravenous methylprednisolone pulse therapy followed by rituximab induction, leading to rapid clinical improvement and stabilization. He remained asymptomatic during follow‐up. This case highlights an extremely rare presentation of GPA with pancreatic mass and diffuse alveolar hemorrhage, emphasizing the importance of considering vasculitis in atypical pancreatic lesions with systemic manifestations to enable early diagnosis and appropriate immunosuppressive therapy.

## Key Clinical Message

Granulomatosis with polyangiitis should be considered in patients presenting with pancreatic mass lesions accompanied by systemic features such as hematuria or pulmonary involvement. Early recognition of vasculitis can prevent misdiagnosis as malignancy, avoid unnecessary surgery, and enable timely initiation of life‐saving immunosuppressive therapy.

## 1 Introduction

Granulomatosis with polyangiitis (GPA) is a rare necrotizing antineutrophil cytoplasmic antibody‐associated vasculitis characterized by inflammation in small‐sized arteries. GPA often presents with nonspecific constitutional symptoms and commonly involves a triad of (i) symptoms from the upper (nasal obstruction, sinusitis, and crusting rhinitis) and lower respiratory tract (lung nodules and alveolar hemorrhage), (ii) systemic vasculitis, and (iii) kidney involvement (necrotizing glomerulonephritis). Gastrointestinal tract involvement is a rather rare entity in systemic vasculitis, especially GPA, and is often associated with poor prognosis. Pancreatic disease is even rarer [1]. Here we present the extremely rare case of GPA presenting as a pancreatic mass and diffuse alveolar hemorrhage.

## 2 Case Presentation

### 2.1 Case History and Examination

A 44‐year‐old male was referred to our center for evaluation of acute respiratory distress during preoperative admission for a suspected pancreatic malignancy. The patient had initially been evaluated at another facility for abdominal discomfort, where imaging revealed a pancreatic tail mass, and he was subsequently planned for pancreatectomy.

One day prior to referral, he developed acute onset of fever with chills and rigors, cough with approximately one teaspoon of blood‐streaked sputum per episode, and progressive shortness of breath. There was no history of chest pain, weight loss, night sweats, alcohol use, or smoking. He had no known comorbidities and no prior history of tuberculosis or autoimmune disease.

On admission, the patient was conscious, oriented, and cooperative. He was tachypneic (respiratory rate: 28 breaths/min), tachycardic (heart rate: 112 beats/min), and pale, with an oxygen saturation of 96% on room air. Blood pressure and temperature were within normal limits. Cardiovascular and neurological examinations were unremarkable. Respiratory examination revealed bilateral basal crepitations.

### 2.2 Differential Diagnosis, Investigation and Treatment

Given the acute presentation, the initial working diagnosis included:

- Severe community‐acquired pneumonia
- Pulmonary hemorrhage syndromes (including vasculitis)
- Pulmonary tuberculosis
- Malignancy‐related pulmonary involvement

The patient met criteria for severe community‐acquired pneumonia (based on IDSA minor criteria), and empirical intravenous antibiotics (piperacillin–tazobactam and meropenem) were initiated along with supportive oxygen therapy (5 L/min via face mask). Hydrocortisone was also administered.

Initial laboratory investigations revealed severe anemia (hemoglobin 7.6 g/dL), marked leukocytosis (30,150/mm^3^), and thrombocytosis (747,000/mm^3^). Inflammatory markers were elevated. Blood and sputum cultures were negative.

Urinalysis demonstrated significant abnormalities, including proteinuria (++), hematuria, and numerous red blood cells with dysmorphic morphology (46%), along with occasional pus cells suggestive of underlying glomerular involvement.

Chest radiography showed bilateral multilobar heterogeneous opacities (Figure 1). High‐resolution computed tomography (HRCT) of the chest demonstrated diffuse ground‐glass opacities with areas of consolidation (Figure 2), findings highly suggestive of diffuse alveolar hemorrhage rather than infective consolidation.

**FIGURE 1:**
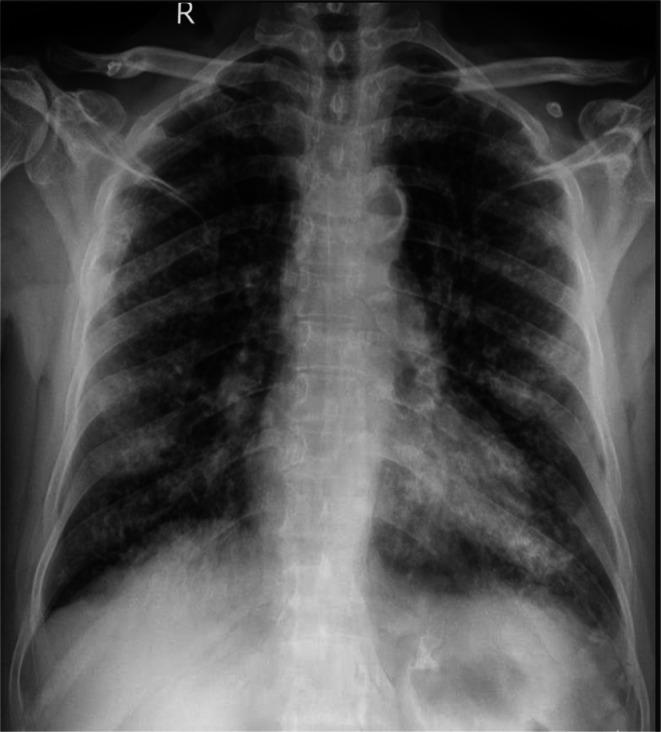
Chest X‐RAY posterior–anterior view showing the bilateral heterogeneous opacities.

**FIGURE 2:**
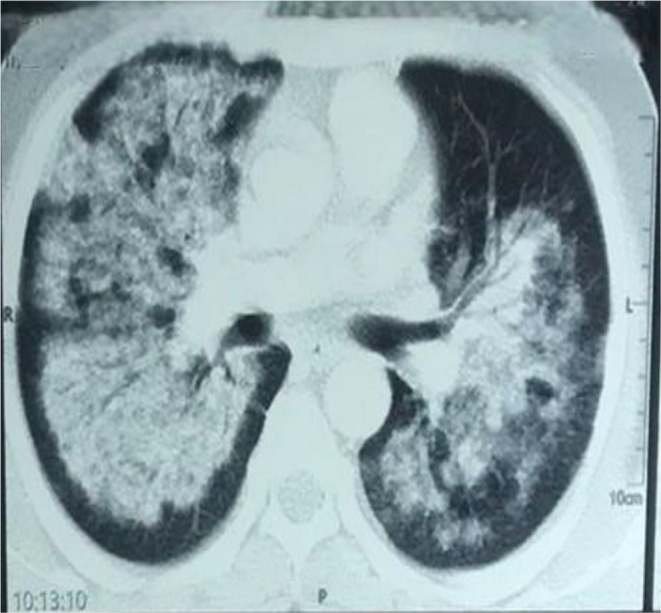
CECT chest showing bilateral diffuse ground glass opacities and consolidations likely diffuse alveolar hemorrhage.

In parallel, further evaluation of the pancreatic lesion was performed. Abdominal ultrasonography revealed a nodular heteroechoic lesion in the left splenorenal region. Contrast‐enhanced computed tomography (CECT) of the abdomen showed an asymmetrically enlarged pancreatic tail with a small hyperdense focus (7 mm) and associated splenic vein obliteration (Figure 3). Magnetic resonance imaging (MRI) demonstrated altered signal intensity in the pancreatic tail and distal body, raising suspicion for a neoplastic process (Figure 4).

**FIGURE 3:**
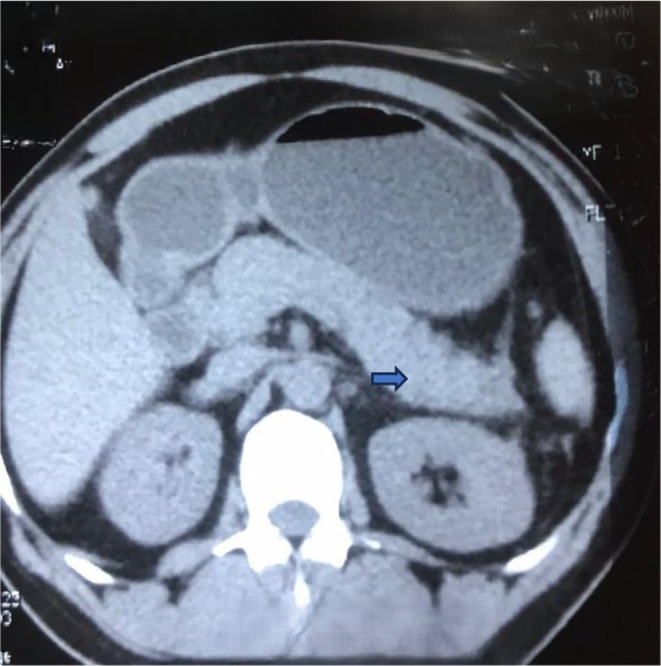
Contrast CT abdomen showing asymmetrical bulky pancreatic tail with nodular iso to hypodense area.

**FIGURE 4:**
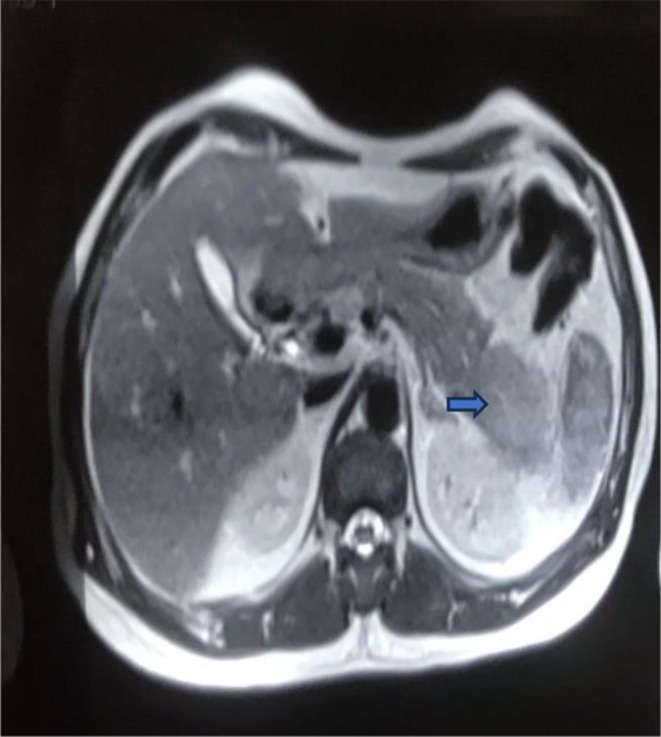
MRI T1W1 sequence of pancreas showing altered signal in tail and distal part of body of pancreas.

Despite broad‐spectrum antibiotic therapy, the persistence of hemoptysis, severe anemia disproportionate to infection, characteristic radiological findings of alveolar hemorrhage, and concurrent urinary abnormalities prompted reconsideration of the diagnosis toward a systemic process.

Subsequent serological testing revealed strongly positive cytoplasmic antineutrophil cytoplasmic antibodies (c‐ANCA) with a titer of 105 RU/mL. Antinuclear antibody (ANA) testing was negative, and complement levels (C3, C4) were within normal limits.

Based on the combination of:

- Diffuse alveolar hemorrhage.
- Evidence of glomerulonephritis (hematuria with dysmorphic red blood cells and proteinuria).
- Strongly positive c‐ANCA.

A diagnosis of ANCA‐associated vasculitis, most consistent with granulomatosis with polyangiitis, was established.

Tissue diagnosis was considered; however, bronchoscopy and fine‐needle aspiration cytology (FNAC) of the pancreatic lesion were deferred initially due to the patient's acute clinical instability with respiratory compromise. Following stabilization, these procedures were recommended but were declined by the patient and his family due to financial constraints and significant clinical improvement. The risks and benefits of invasive diagnostic procedures were carefully discussed with the patient.

Following confirmation of the diagnosis, intravenous methylprednisolone pulse therapy was initiated, followed by rituximab for induction of remission. The patient showed marked clinical improvement, with resolution of respiratory symptoms and stabilization of hemodynamic parameters within 10 days of intensive care unit stay. Oxygen support was gradually weaned, and hemoglobin levels improved with supportive care.

### 2.3 Outcome and Follow‐Up

The patient was subsequently transferred to the general ward, where he remained clinically stable for an additional 5 days before discharge. He was advised regular follow‐up and further evaluation of the pancreatic lesion with FNAC and bronchoscopy.

At follow‐up visits conducted at 3‐month intervals, the patient remained asymptomatic with no recurrence of respiratory or abdominal symptoms. Despite repeated counseling, the patient declined invasive diagnostic procedures. On subsequent telephonic follow‐up, he continued to report good health.

## 3 Discussion

Granulomatosis with polyangiitis (GPA) is a necrotizing small‐vessel vasculitis belonging to the spectrum of antineutrophil cytoplasmic antibody (ANCA)‐associated vasculitides, typically involving the respiratory tract and kidneys [2, 3, 4, 5]. Although pulmonary and renal manifestations are well recognized, gastrointestinal involvement is uncommon, and pancreatic disease represents an exceedingly rare manifestation [2]. The present case illustrates an unusual presentation of GPA as a pancreatic mass mimicking malignancy, in association with diffuse alveolar hemorrhage (DAH).

Pancreatic involvement in GPA has been reported only in isolated case reports and small series, often presenting as mass‐like lesions or pancreatitis [1, 2]. Such lesions can closely resemble pancreatic malignancy radiologically, leading to potential misdiagnosis and unnecessary surgical intervention. In our patient, a pancreatic mass initially raised suspicion for malignancy, and surgical management was planned. However, the emergence of systemic features prompted reconsideration of the diagnosis. Although histopathological confirmation was not obtained, the rapid clinical and radiological improvement following immunosuppressive therapy strongly supports the diagnosis of a vasculitic inflammatory pseudotumor. Similar outcomes have been reported in prior cases where pancreatic lesions resolved after initiation of immunosuppressive therapy [2].

Diffuse alveolar hemorrhage is a severe and potentially life‐threatening complication of ANCA‐associated vasculitis, resulting from pulmonary capillarity's [6, 7]. Clinically, it is characterized by hemoptysis, anemia, and diffuse pulmonary infiltrates, although hemoptysis may be absent in some patients [6]. In our case, the presence of hemoptysis, severe anemia, and bilateral ground‐glass opacities on high‐resolution computed tomography was consistent with DAH. Importantly, DAH is associated with significantly increased mortality in GPA and requires prompt recognition and aggressive immunosuppressive therapy [7, 8].

A key diagnostic challenge in this case was the initial presentation mimicking severe community‐acquired pneumonia. The patient met clinical criteria for severe infection and was empirically treated with broad‐spectrum antibiotics. However, several findings prompted a shift toward a rheumatologic etiology: persistent hemoptysis with disproportionate anemia, radiological features suggestive of alveolar hemorrhage rather than infection, urinary abnormalities including dysmorphic red blood cells and proteinuria indicating glomerulonephritis, and strongly positive c‐ANCA. This diagnostic transition highlights the importance of considering systemic vasculitis in patients with multiorgan involvement and atypical clinical progression [9, 10].

The diagnosis of GPA in this patient was established based on a combination of clinical, radiological, and serological findings. While tissue biopsy remains the gold standard, current guidelines recognize that a diagnosis can be made using surrogate markers in appropriate clinical settings, particularly in the presence of positive PR3‐ANCA (c‐ANCA) and characteristic organ involvement [9, 10, 11]. In our case, biopsy procedures including bronchoscopy and fine‐needle aspiration cytology were initially deferred due to clinical instability and subsequently declined by the patient due to financial constraints and clinical improvement. This represents an important limitation, and pancreatic involvement remains presumptive. Nevertheless, the constellation of findings and the excellent response to immunosuppressive therapy strongly support the diagnosis.

Management of severe GPA involves induction therapy with high‐dose glucocorticoids in combination with either cyclophosphamide or rituximab [11, 12, 13, 14]. Rituximab has emerged as an effective alternative with comparable efficacy and a favorable safety profile, particularly in patients with severe or relapsing disease [10, 11, 12]. Methotrexate has been considered an option for patients without organ‐threatening GPA, but relapse rates were high and most patients subsequently required cyclophosphamide [15, 16]. In this case, rituximab was selected due to the presence of life‐threatening DAH and the need for rapid disease control. The patient demonstrated marked clinical improvement within 10 days, consistent with previous reports suggesting rapid resolution of pulmonary manifestations with rituximab‐based therapy [3, 7]. This favorable response further supports the inflammatory vasculitic nature of both pulmonary and pancreatic lesions.

This case highlights several important clinical implications. First, GPA should be considered in the differential diagnosis of pancreatic mass lesions, particularly when accompanied by systemic features such as pulmonary or renal involvement. Second, early recognition of DAH is crucial, as timely initiation of immunosuppressive therapy can significantly reduce mortality. Third, reliance solely on imaging findings may lead to misdiagnosis; integration of clinical, laboratory, and serological data is essential for accurate diagnosis. Finally, in selected cases, a diagnosis of GPA can be reasonably established without histopathological confirmation when supported by strong clinical and serological evidence.

## 4 Conclusion

Granulomatosis with polyangiitis is a multisystem autoimmune vasculitis with diverse clinical manifestations. While pulmonary and renal involvement are well recognized, pancreatic mass presentation is exceedingly rare and may mimic malignancy, leading to diagnostic delays and unnecessary surgical planning. The coexistence of diffuse alveolar hemorrhage further underscores the severity of the disease and the need for urgent intervention.

This case emphasizes the importance of comprehensive clinical evaluation, integration of imaging findings, and ANCA serology in establishing the diagnosis when tissue biopsy is not feasible. Rituximab‐based induction therapy, combined with high‐dose corticosteroids, resulted in rapid clinical improvement and sustained remission.

Awareness of such atypical presentations is crucial to facilitate early diagnosis, initiate timely immunosuppressive therapy, and improve patient outcomes.

## Author Contributions

**Anil Shahi:** conceptualization, formal analysis, visualization. **Ayush Neupane:** conceptualization, investigation, supervision. **Bishaka Adhikari:** conceptualization, software, supervision, visualization, writing – original draft, writing – review and editing.

## Funding

The authors have nothing to report.

## Consent

Human subjects: Written informed consent was obtained from the patient's parents/legal guardian for publication and any accompanying images. A copy of the written consent is available for review by the Editor‐in‐Chief of this journal on request.

## Conflicts of Interest

The authors declare no conflicts of interest.

## Data Availability

The data supporting the findings of this study are included within the article. Additional patient‐level data are not publicly available due to privacy and confidentiality considerations but may be available from the corresponding author upon reasonable request and with appropriate ethical approval.
